# Identification of human intestinal parasites affecting an asymptomatic peri-urban Argentinian population using multi-parallel quantitative real-time polymerase chain reaction

**DOI:** 10.1186/s13071-015-0994-z

**Published:** 2015-07-17

**Authors:** Rubén O. Cimino, Rebecca Jeun, Marisa Juarez, Pamela S. Cajal, Paola Vargas, Adriana Echazú, Patricia E. Bryan, Julio Nasser, Alejandro Krolewiecki, Rojelio Mejia

**Affiliations:** Instituto de Investigaciones en Enfermedades Tropicales - Universidad Nacional de Salta/CONICET, Oran, Argentina; Cátedra de Química Biológica, Facultad de Ciencias Naturales, Universidad Nacional de Salta, Oran, Argentina; National School of Tropical Medicine, Baylor College of Medicine, Houston, TX USA; Fundación Mundo Sano, Buenos Aires, Argentina

**Keywords:** Intestinal parasites, Molecular diagnostics, Prevalence, Argentina, Hookworm speciation, Real-time PCR

## Abstract

**Background:**

In resource-limited countries, stool microscopy is the diagnostic test of choice for intestinal parasites (soil-transmitted helminths and/or intestinal protozoa). However, sensitivity and specificity is low. Improved diagnosis of intestinal parasites is especially important for accurate measurements of prevalence and intensity of infections in endemic areas.

**Methods:**

The study was carried out in Orán, Argentina. A total of 99 stool samples from a local surveillance campaign were analyzed by concentration microscopy and McMaster egg counting technique compared to the analysis by multi-parallel quantitative real-time polymerase chain reaction (qPCR). This study compared the performance of qPCR assay and stool microscopy for 8 common intestinal parasites that infect humans including the helminths *Ascaris lumbricoides, Ancylostoma duodenale, Necator americanus, Strongyloides stercoralis, Trichuris trichiura,* and the protozoa *Giardia lamblia, Cryptosporidium parvum/hominis,* and *Entamoeba histolytica,* and investigated the prevalence of polyparasitism in an endemic area.

**Results:**

qPCR showed higher detection rates for all parasites as compared to stool microscopy except *T. trichiura*. Species-specific primers and probes were able to distinguish between *A. duodenale* (19.1 %) and *N. americanus* (36.4 %) infections. There were 48.6 % of subjects co-infected with both hookworms, and a significant increase in hookworm DNA for *A. duodenale* versus *N. americanus* (119.6 fg/μL: 0.63 fg/μL, *P <* 0.001) respectively. qPCR outperformed microscopy by the largest margin in *G. lamblia* infections (63.6 % versus 8.1 %, *P* < 0.05). Polyparasitism was detected more often by qPCR compared to microscopy (64.7 % versus 24.2 %, *P* < 0.05).

**Conclusions:**

Multi-parallel qPCR is a quantitative molecular diagnostic method for common intestinal parasites in an endemic area that has improved diagnostic accuracy compared to stool microscopy. This first time use of multi-parallel qPCR in Argentina has demonstrated the high prevalence of intestinal parasites in a peri-urban area. These results will contribute to more accurate epidemiological survey, refined treatment strategies on a public scale, and better health outcomes in endemic settings.

## Background

The World Health Organization (WHO) classifies most parasitic diseases as Neglected Tropical Diseases (NTD) [1]. Soil-transmitted helminths (STH) and intestinal protozoa are distributed widely throughout the world and are more prevalent in tropical and sub-tropical regions. STH (*Ascaris lumbricoides, Ancylostoma duodenale, Necator americanus, Strongyloides stercoralis,* and *Trichuris trichiura*) affect more than 2 billion people worldwide [2]. These species produce a wide array of symptoms, from asymptomatic (not reporting intestinal complaints) to including diarrhea, abdominal pain, general malaise, and weakness that may impact learning capacities and impaired physical growth [3–5]. Hookworms cause chronic intestinal blood loss that results in anemia, significantly impacting health [6]. Infections with pathogenic intestinal protozoa infections, primarily *Giardia lamblia* and *Entamoeba histolytica*, are also of considerable public health importance. In northern Argentina, mainly in the Yungas rainforest and Chaco regions, recent reports indicate prevalence rates of over 20 %, with some areas approaching 50 % [7, 8].

The standard diagnostic method for gastrointestinal parasites is direct microscopic examination of stool samples. For STH infections, the recommended technique for microscopic diagnosis is Kato-Katz, except for *S. stercoralis*, for which the Baermann, agar plate, and Harada Mori methods are recommended [9]. Other fecal egg count (FEC) techniques such as McMaster and the recently developed FLOTAC and Mini-FLOTAC have also been successfully used for diagnosis of STH infections in humans [10, 11].

Recently, molecular tools such as quantitative real-time PCR (qPCR) have improved diagnosis of gastrointestinal parasites [12, 13]. Multi-parallel and multiplex qPCR have the advantage of enabling detection of multiple parasite species using a single stool sample as well as the ability to determine the intensity of infection of each species [12, 13]. This study, the first use of multi-parallel qPCR in Argentina, sought to compare the performance of multi-parallel qPCR assay and stool microscopy in an endemic area for 8 common intestinal parasites including the intestinal helminths *Ascaris lumbricoides, Ancylostoma duodenale, Necator americanus, Trichuris trichiura, Strongyloides stercoralis* and the protozoa *Cryptosporidium parvum/hominis, Entamoeba histolytica,* and *Giardia lamblia.* Of particular interest is the diversity of intestinal parasite infections within the study site, Orán, Argentina, as it is a peri-urban locale with higher population density than a rural area, but fewer community services such as sanitation and paved roads than an urban center. Although qPCR has previously been compared to stool microscopy in Ecuador [12], it was important to field test qPCR at this new site in Argentina. Especially since McMasters method is the diagnostic standard for microscopy in Argentina and has never been previously compared to qPCR.

This study also investigated the prevalence of polyparasitism and the differences in the epidemiology of hookworm infection. Hookworm infection is of special interest from a diagnostic perspective because infection can result in anemia and childhood stunting [2–6]. Stool microscopy cannot distinguish between *Necator americanus* and *Ancylostoma duodenale* ova, while qPCR can differentiate species and co-infections without waiting for larvae development [12].

## Methods

### Ethics, consent and permissions

This study was approved by the internal review boards of Baylor College of Medicine and Universidad Nacional de Salta Argentina.

Informed written consent was obtained from each participant or from a parent/guardian. Anti-parasitic treatment, based on microscopy findings, was provided per standard of care in the region. The majority of subjects were of preschool age and did not receive standard mass drug administration of anthelmintics. Study design and protocols were approved by the bioethics committee of Universidad Nacional de Salta, Argentina.

### Sample collection

Fecal samples were collected from a random cohort of 99 asymptomatic subjects ages 7 months through 57 years old (geometric mean, 7.2 years old) living in the Orán department, Salta Province, Argentina. Samples were collected from September through December 2012. Stool was then frozen without fixatives at−80 °C until used for DNA extraction and retrospective study with qPCR.

### Microscopy

Stool samples were examined by Telemann concentration, as previously described, and McMaster methods at the time of stool collection [14]. The McMaster method was performed following the standard procedure: 2 g of feces were filtered and homogenized with 30 ml of saturated saline. Two flotation chambers (1 ml each) were filled for each sample. Eggs were allowed to float for 3 min then counted. This number was multiplied by 50 for the egg per gram count (epg). qPCR was compared to any positive microscopy sample using the concentration and McMaster method.

### DNA extraction

DNA was extracted from 50 mg stool by using the FastPrep® Spin Kit for Soil (MP Biomedicals, Santa Ana, CA) according to manufacturer’s instructions for all parasites except *T. trichiura.* An additional step required for the extraction of *T. trichiura* DNA was performed as previously described [12]. All samples were processed in Salta, Argentina.

### Multi-parallel qPCR

Multi-parallel qPCR was performed as previously described [12] with the same species-specific primers and FAM-labeled minor groove binder probes (Applied Biosystems, Foster City, CA). Samples were run on an ABI ViiA7 PCR in Houston, Texas with default parameters and 40 cycles. DNA concentrations were calculated using a standard curve.

### Statistical analysis

All statistical analyses were performed with Prism version 6.0f (GraphPad, La Jolla, CA). Comparisons of microscopy with qPCR were analyzed by using Fisher’s exact test, and *P* values <0.05 were considered statistically significant. Correlations were estimated by calculation of Spearman’s rank correlation coefficients. Welch’s unpaired t-test was used in comparisons of DNA concentrations.

## Results

### Comparison of qPCR with microcopy

The results of qPCR and microscopy for all studied intestinal parasites are summarized in Table 1. qPCR was significantly more sensitive than stool microscopy in identifying *S. stercoralis* (21.2 % versus 3.0 %, *P* < 0.05)*, G. lamblia* (63.6 % versus 8.1 %, *P* < 0.05)*,* and total hookworm infections (37.4 % versus 21.2 %, *P* < 0.05). A stark difference was noted with *G. lamblia* as qPCR detected more positive samples than stool microscopy by a factor of 8 with a sensitivity of 87.5 and 97.2 % negative predictive value (NPV). Both methods identified similar number of positive samples of *A. lumbricoides,* but qPCR detected three more than concentration microscopy and nine more than McMaster method. For *A. lumbricoides,* qPCR had a 91.3 % sensitivity and 90.5 % NPV. For *S. stercoralis,* qPCR had a 100 % sensitivity and NPV.

**Table 1:** Comparison of intestinal parasite detection by stool qPCR and microscopy in 99 subjects

Parasite	No. positive by multi-parallel qPCR (%)	No. positive by concentration microscopy (%)	No. positive by McMaster microscopy (%)	DNA concentrations (fg/μL) in stool positive by microscopy, median (range)	DNA concentrations (fg/μL) in stool negative by microscopy, median (range)
*Ascaris lumbricoides*	56/99 (56.5)	53/99 (53.5)	47/99 (47.5)	1.02 (0.001 – 42.3)	0.48 (0.003–10.431)
*Cryptosporidium parvum/hominis*	0/99 (0)	NA	NA	NA	NA
*Ancylostoma duodenale*	19/99 (19.1)	NA	NA	55.1 (5.87–278.2)^a^	106.9 (26.2–384.2)
*Necator americanus*	36/99 (36.4)	NA	NA	12.5 (0.024–278.2)^a^	1.59 (0.001–239.5)
Hookworm*	37/99 (37.4)	21/99 (21.2)^a^	21/99 (21.2)^a^	NA	NA
Strongyloides stercoralis*	21/99 (21.2)	3/99 (3.0)	NA	65.9 (14.7–123.6)	0.22 (0.0009–48.6)
Giardia lamblia*	63/99 (63.6)	8/99 (8.1)	NA	5.27 (0.02–1847.4)	0.27 (0.007–5697.8)
*Entamoeba histolytica*	1/99 (1.0)	0/99 (0)	NA	0.003	0
*Trichuris trichiura*	1/99 (1.0)	4/99 (4.0)	6/99 (6.1)	0.001	0

*NA* Not applicable

*Indicates statistically significant difference (*P* < 0.05)

^a^Microscopy is unable to distinguish between the two hookworm species, *A. duodenale* and *N. americanus*

Additionally, qPCR has the ability to distinguish *A. duodenale* and *N. americanus. N. americanus* (36.4 %) was more prevalent in this study population than *A. duodenale* (19.1 %)*.* Significantly more infections with combined hookworm species were detected with qPCR compared to concentration and McMaster method (37.4 % versus 21.2 %, *P* < 0.05), respectively and had a 95.5 % sensitivity and 98.4 % NPV.

The only intestinal parasite that stool microscopy detected more frequently, was *T. trichiura* though the difference was not significant (*P =* 0.118). No *Cryptosporidium parvum/hominis* positive samples were identified in the study population by qPCR. Only one *E. histolytica* positive sample was detected by qPCR though it was not seen by microscopy. DNA concentrations were higher in those samples also found to be positive by microscopy except in the case of *A. duodenale,* but did not reach statistical significance*.*

### Hookworm studies

**Fig. 1:**
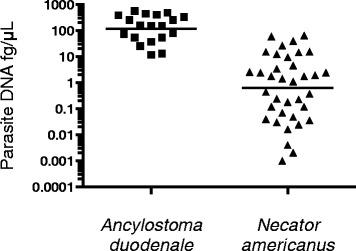
DNA concentrations (fg/μL) of two hookworm species *A. duodenale* (■) and *N. americanus* (▲) by qPCR. Higher DNA concentration of *A. duodenale* 119.6 fg/μL versus 0.63 fg/μL for *N. americanus* (*P <* 0.001)

Since qPCR has the ability to distinguish between hookworm species, *A. duodenale* and *N. americanus,* this study assessed the DNA concentration found in positive samples of both species (Fig. 1). The DNA concentration was significantly higher with *A. duodenale* as compared to *N. americanus* (geometric mean of *A. duodenale*: 119.6 fg/μL versus *N. americanus*: *0*.63 fg/μL, *P <* 0.001). There were 18/37 (48.6 %) samples containing both *A. duodenale* and *N. americanus* DNA, without any correlation between the two species’ DNA concentrations (*P >* 0.05).

**Fig. 2:**
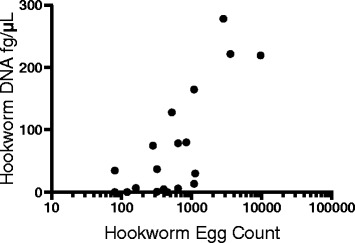
Correlation of hookworm DNA concentration (fg/μL) with hookworm egg count by McMaster method (eggs per gram) (*r* = 0.632, *P* = 0.0028). Only samples positive by qPCR are shown

Comparison of hookworm egg counts by the McMaster method with hookworm DNA concentration (geometric mean) in samples positive by both methods showed a statistically significant correlation (Fig. 2, *r* = 0.632, *P =* 0.0028). The average DNA concentration of *A. duodenale* and *N. americanus* in co-infected individuals was used in this correlation (Fig. 2).

### Detecting polyparasitism by qPCR

**Fig. 3:**
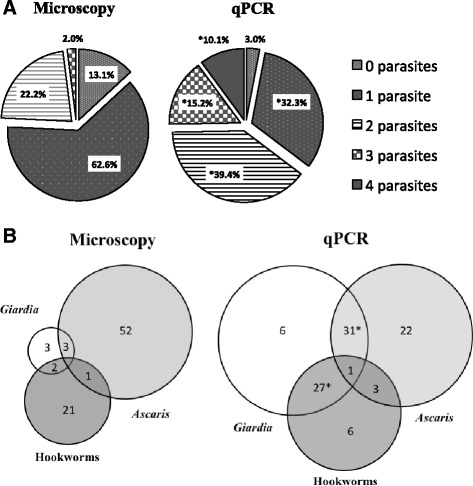
Detection of polyparasitism by quantitative multi-parallel real-time polymerase chain reaction (qPCR) and microscopy in 99 patients from Argentina. **a** Percentages of stools positive for 0–4 parasites by microscopy or qPCR. *Indicates statistically significant difference (*P <* 0.05). **b** Increased detection of the three most prevalent intestinal parasite infections (in a Venn diagram) by microscopy and qPCR

The vast majority of the study population had positive stool samples for at least one kind of intestinal parasite infection by qPCR and/or microscopy (96/99, 97.0 %). Microscopy was only able to detect two or more intestinal parasite species in 24/99 (24.2 %) samples as compared to 64/99 (64.7 %) samples identified by qPCR (Fig. 3a). Microscopy distinguished a maximum of three different intestinal parasites in the same sample (2/99, 2.0 %) compared to 15/99 (15.2 %) for qPCR. While qPCR was even able to detect up to four different species in the same samples (10/99, 10.1 %), microscopy did not detect any. All polyparasitic comparisons were statistically significant (*P* < 0.05).

In terms of specific co-infections, qPCR identified more cases of co-infection by *G. lamblia/A. lumbricoides* (32 versus 3, *P* < 0.05)*, G. lamblia/*hookworm (28 versus 2, *P* < 0.05), and *A. lumbricoides/*hookworm (4 versus 1, *P* > 0.05) than microscopy, respectively (Fig. 3b). *G. lamblia/A. lumbricoides* was the most commonly encountered co-infection by both qPCR and microscopy though qPCR detected more samples by a factor of 10. *G. lamblia* and *A. lumbricoides* were also the most prevalent species in the study population by both microscopy and qPCR. The same four parasites (*G. lamblia, S. stercoralis, N. americanus,* and *A. duodenale*) were found in all ten samples that were positive by qPCR for a quadruple infection.

## Discussion

The geographic distribution of intestinal parasite infections overlaps significantly with resource-poor nations that lack consistent access to clean water, sanitation, and health care [15]. Reducing morbidity associated with intestinal parasites requires reliable data on prevalence. This is difficult to accomplish when the diagnostic standard, stool microscopy, often lacks sensitivity and specificity. Multi-parallel qPCR was developed to improve upon the diagnostic sensitivity of microscopy, especially in epidemiological studies, and to complement microscopy as a diagnostic tool in resource-limited settings. This study compared qPCR assay to microscopy in the diagnosis of 8 common intestinal parasites. Particularly, the degree of hookworm infection and the prevalence of polyparasitism were investigated.

Extremely high rates of intestinal parasite infections were detected, both by microscopy and qPCR. Although, qPCR detected a higher number of total infections, the high rates of infection were especially noteworthy in an asymptomatic population. qPCR detected significantly more hookworm, *S. stercoralis,* and *G. lamblia* infections than microscopy. qPCR and microscopy performed similarly for *A. lumbricoides,* likely because the eggs are more easily visualized than other species. For STH, the prevalence of ascariasis and hookworm infection by qPCR exceeds the estimated national prevalence of 5–24.9 % [16]. Consideration should also be given to geographical region, since temperature, area, and local populations have varied parasitosis prevalence [17]. There were none or low rates of *C. parvum/hominis*, *E. histolytica,* and *T. trichiura* identified by both qPCR and microscopy*. T. trichiura* was the only species for which microscopy detected more positive samples which may suggest loss of yield in the extra step of processing required in the qPCR protocol. Since qPCR has previously been shown to be more sensitive than microscopy in detecting *T. trichiura,* lack of primer specificity is likely not the issue [12]. Another possibility for this discordance is subtle genetic differences in the ITS-1 target region of *Trichuris trichiura* found in Argentina. Primer/probe combinations were field tested and optimized for use in Ecuador [12]. This may explain the lack of qPCR sensitivity and further research is needed.

A benefit of this qPCR assay is the ability to distinguish between the two hookworm species, *A. duodenale* and *N. americanus*. The hookworm species have long been grouped together in discussion since treatment is the same and it is very laborious to rear larvae to distinguish the two species. However, identifying the predominant species, *N. americanus* in this study, can contribute to better control measures as *N. americanus* transmission is frequent at defecation sites, because it cannot survive for long periods of time in the environment like *A. duodenale* [18]. The DNA concentration of *A. duodenale* in stool samples was higher than with *N. americanus.* This is consistent with the higher egg output per female *A. duodenale* worm per day [18]. *A. duodenale* has also been associated with more severe symptoms and greater blood loss with increased anemia [19]. This has important implications for anemia and long-term morbidity when higher *A. duodenale* DNA concentrations in asymptomatic individuals are discovered. A study of hookworm infection in Malawian pre-school children used real-time PCR C_t_ values as a proxy for infection load and found an association between increased load of *A. duodenale* and iron deficiency though the sample size was limited [20]. These differences and the lack of data on post-treatment efficacy stratified by hookworm species support the need for better characterization of prevalence by methods such as the qPCR assay [21].

Another benefit of qPCR is the ability to detect helminth DNA in stool without the presence of eggs. Since intestinal worms are continuously shedding and releasing DNA, amplification of DNA from stool is possible and increases parasite detection. However, most DNA from stool samples is overwhelmingly derived from the high density of eggs.

Results from this study also showed that qPCR is able to detect multiple parasite species at a higher rate than microscopy. The overwhelming majority of the study population tested positive for at least two different infections by qPCR. At most, microscopy detected a maximum of three species in the same sample, while qPCR was able to detect four. This has important population health implications for the implementation of mass drug administrations and for decisions related to choosing appropriate anti-parasitic drugs based on more accurate qPCR epidemiological data [22]. This study determined that *G. lamblia* has a high prevalence in this population, but might not be addressed with traditional community-based mass drug administrations that tend to target soil-transmitted helminths [22, 23]. Giardiasis commonly occurred with Ascariasis and hookworm species in dually infected subjects. The data on co-infection with *A. lumbricoides* or hookworms and *G. lamblia* is conflicting, with both positive and negative associations described in the literature [24, 25]. This could be attributed to the differences in water supply and hygiene in the different locations studied. Modulation of immune response in polyparasitism, in which infection with one parasite alters the immune response to another, has also been described and further supports the need for accurate diagnosis of polyparasitism in a population when planning interventions like a hookworm vaccine [25–27].

### Study limitations

A limitation of the study was in the comparison of the hookworm egg count and the hookworm DNA concentration (Fig. 2). Since the ova of the two species are not distinct enough to be distinguishable by microscopy, it is difficult to estimate the egg output with co-infections also complicating the correlation. However, it is meant as a broad overview of the relationship of egg count and DNA concentration found in the stool samples. Another limitation of this study is the inability to determine whether qPCR is detecting residual DNA from previous infections and true prevalence in an endemic area is being overestimated. However, the constant peristalsis of the intestinal tract promotes evacuation of dead parasites and decreases the false positive rates detected by qPCR.

As in a previous Ecuador study, an internal control was not available [12]. This increases the possibility of missing positive samples due to inhibition or errors in extraction. The results of this pilot project did not appear to be impacted by PCR inhibition, given the general increase in detection by qPCR, as seen in previous work [12].

## Conclusions

As the use of molecular diagnostics in determining intestinal parasitic infections has become more common, multi-parallel qPCR assay offers a broad coverage of the most common intestinal parasites including the soil-transmitted helminths and pathogenic protozoa. Not including the cost of the instrument, this assay is less expensive than microscopy and requires less sophisticated supplies than multiplex assays so it can be effectively transferred to relatively resource-limited areas as previously demonstrated [12]. This high-throughput technique is designed to operate in a centralized location, such as a university or local hospital, and can be useful in targeting the community as a public health platform to actively survey current trends in parasitic infections and polyparasitism. Since 30 % of the population worldwide are infected with intestinal parasites that can be associated with developmental delays, stunted growth, and decreased work productivity, there is ample opportunity for the use of this assay as a powerful tool in epidemiological studies [2–5].

This is the first time use of multi-parallel qPCR in Argentina and has demonstrated the high prevalence of intestinal parasites in a peri-urban area. Multi-parallel qPCR will be implemented to further investigate the relationships between different intestinal parasites that occupy the same anatomic niche and their impact on morbidity.
